# Variations in Some Features of Oral Health by Personality Traits, Gender, and Age: Key Factors for Health Promotion

**DOI:** 10.3390/dj12120391

**Published:** 2024-12-03

**Authors:** Allexey Martínez Fuentes, Tania Romo-González, Israel Huesca-Domínguez, Yolanda Campos-Uscanga, Antonia Barranca-Enríquez

**Affiliations:** 1 Laboratorio de Biología y Salud Integral, Instituto de Investigaciones Biológicas, Universidad Veracruzana, Xalapa 91190, Mexico; allmartinez@uv.mx; 2 Instituto de Investigaciones Biológicas, Universidad Veracruzana, Xalapa 91190, Mexico; ishuesca@uv.mx; 3 Instituto de Salud Pública, Universidad Veracruzana, Xalapa 91190, Mexico; ycampos@uv.mx; 4 Centro de Estudios y Servicios en Salud, Universidad Veracruzana, Veracruz 91700, Mexico

**Keywords:** integrative health, well-being, caries, parafunctional habits, gender and age

## Abstract

**Background**: Oral diseases remain among the most common non-communicable diseases worldwide, affecting almost half of the world’s population. This is partly because there has been a separation of the mouth from the rest of the body and human health, and psychological aspects such as personality, thoughts, and emotions are not taken into account in the dental office. The objective was to analyze the relationship between oral health conditions and personality traits in adult patients who underwent dental treatment at the Center for Health Studies and Services. **Methods**: This was a descriptive, observational, and correlational study, carried out at the Center for Health Studies and Services. A total of 184 patients who attended the dentistry area in the period from October 2022 to May 2023 participated in the study, of which 59.78% were women and 40.21% men. The age of the population was 18 to 79 years, with the age range of 21–40 years being the most prevalent (48.37%). **Results**: The results show that although the hygiene of the population treated was good (0.77 ± 0.79) and that the perception of oral health was positive (14.34 ± 9.43), the means and percentages of oral pathologies and parafunctional habits were high (i.e., DMFT: 9.98 ± 5.40; attrition: 87.50%; onychophagia: 45.10%). It is noteworthy that both the correlation, network, multiple line regression, and logistic regression analyses showed associations of the personality, gender, and age variables with a history of caries and oral hygiene as well as with parafunctional habits. **Conclusions**: Therefore, variations in both the personality and the age and gender of the patients treated have repercussions on oral health conditions, which can be used in the prevention of oral diseases and in health promotion.

## 1. Introduction

The World Health Organization (WHO) has long defined oral health as an oral state that is free from disease and contributes to the normal function of the mouth [1]. However, given that research has shown the importance it has for general health, and even more so that it is inseparable from it, in 2022, the WHO reformulated its definition as follows:

“Oral health is the state of the mouth, teeth, and orofacial structures that enables individuals to perform essential functions such as eating, breathing, and speaking, and encompasses psychosocial dimensions such as self-confidence, well-being and the ability to socialize and work without pain, discomfort and embarrassment. Oral health varies over the life course from early life to old age, is integral to general health and supports individuals in participating in society and achieving their potential” [1].

Despite this great recognition of the multifaceted role that oral health has for the lives of individuals and communities, its impact has been minimal, since oral diseases continue to be among the most common non-communicable diseases worldwide, affecting to almost half of the world’s population. Furthermore, the number of cases worldwide has increased by one billion in the last 30 years, which indicates that for many people, it is impossible to access comprehensive oral rehabilitation [1].

Therefore, the global burden of dental health disorders is increasing, particularly in low- and middle-income countries, since for those who can obtain oral rehabilitation, the costs are usually high and can represent a significant impact on their economy, that is, there are inequalities with severe social and economic consequences that impact the persistence of oral diseases and a greater burden of morbidity observed in vulnerable and highly marginalized communities, among which women are found. Furthermore, the overall burden of oral health disorders on health centers continues to increase due to population growth and aging. In this sense, private and public spending on oral health rehabilitation is reaching almost 390 billion dollars worldwide, with an unequal distribution regionally and between different countries [1].

All this is paradoxical since oral health professionals (dental surgeons, rehabilitators, periodontists, endodontists, etc.) have increased considerably in all countries, so perhaps the lack of success in reducing these diseases is due to the focus on disease care and not on prevention and much less on health promotion. Likewise, fundamental aspects for oral health such as psychosocial factors (i.e., stress and its coping, self-efficacy, sense of coherence, personality, social support, age, or gender) that can alter patterns of behavior related to health and that are directly related to risks to it [2] are not taken into account in the clinic, nor in the training of dental professionals, nor in research [3].

These data are important not only because the consequences of untreated oral diseases (including functional limitations, physical symptoms, and detrimental impacts on mental, emotional, and social well-being) are serious and debilitating, greatly affecting productivity and the ability to work, quality of life, and the social participation, but also because oral health plays a fundamental role in people’s self-esteem and well-being by allowing essential human functions within the physical, psychological, emotional, and social domains to occur [1,4]. Furthermore, the oral cavity has been identified by psychology as the area of the first physiological needs and emotional gratifications; with it, we take a taste of the world around us, since it provides the first sensations of security, pleasure, satisfaction, and success, bringing all this oral activity to the first perceptions of the self [3,5]. In this sense, it is essential to study the links between oral health and psychosocial factors such as personality, age, and gender to expand the understanding of these possible relationships, and based on the results, propose strategies for the prevention of oral diseases and health promotion.

In this regard, the study of the link between personality and health-disease processes was initiated by the Greek doctor Hippocrates when he developed the theory of temperament. This medical theory has its roots in the ancient theory of the four humors (460–370 BC): blood, yellow bile, black bile, and phlegm. Since then, many physicians have greatly developed our modern theories of temperament, however, Hans Eysenck was the first to analyze personality differences using a psychostatistical method, and his research proposed that temperament has a biological basis and consists of personality components of extraversion, psychoticism, and neuroticism. Likewise, in 1990, Digman proposed a five-factor model of personality, which was expanded by Lewis Goldberg. In this model, they considered that personality is divided into components, which are neuroticism (or emotionality), extroversion, agreeableness, conscientiousness, and openness to experience. It is worth noting that while for a long period of time it was believed that personality traits remained immutable and stable, longitudinal research has shown that personality changes significantly as a result of life experiences and lessons acquired in early adulthood [6].

Based on this theory, research has shown that personality features of human beings can influence oral health, and that the impact of the social, behavioral, educational, cultural, economic factors, and lifestyle are also related to the influence of personality characteristics on oral health status [6]. Thus, it is considered that stress, depression in daily life, anxiety, self-esteem, age, gender, and smoking are related to oral health behavior.

In this regard, Thomson et al. [7] suggested that personality may be related to oral health as it increases the risk of oral ailments and changes individuals’ attitude toward the disease. For example, several physical, psychological, and environmental factors have the power to alter gingival tissues and host immune responses, resulting in a more severe expression of periodontal disease. There is also an impact of an individual’s general personality on the person’s oral hygiene status since it has been found that higher scores on the Simplified Oral Hygiene Index and the Gingival Plaque Index correspond to people with a neuroticism-type personality [6]. Likewise, the frequency of visiting the dentist varies with personality, since most people who do not visit the dental clinic have been associated with the extroversion and neuroticism groups [6].

On the other hand, Capsi et al. [8] showed that people with high scores in psychoticism were more aggressive, predisposing them to poor oral health and to the habit of bruxism, while people with low scores in extroversion were more likely to smoke, which affects the periodontium and causes periodontitis. Additionally, studies have suggested that depression, stress, and ineffective coping associated with certain personality types may contribute to the development of periodontal disease and dental caries [9,10,11] while the extroversion and neuroticism personalities were more frequent in middle- and low-income groups, which in turn has been associated with a higher prevalence of oral diseases [6,12].

In Mexico, oral diseases and inequalities in their care are very high in various population groups, particularly when it comes to socioeconomic status and gender. For example, according to the 2021 results of the Epidemiological Surveillance System for Oral Pathologies (SIVEPAB), dental caries affect 92.5% of the population who visit health services, a percentage that also varies according to age and gender, being higher as the years go by and in men. In the case of periodontal disease, approximately 60% of the population studied had some sign of disease, a percentage that also increases with age (except for people over 80 years of age) [13]. In this sense, studying the relationship of personality, gender, and age could be useful in preventive dentistry since brief cognitive interventions can alter the attitudes and values leveraged by personality traits [6]. This is especially considering the international health commitments that Mexico has acquired since 2021, in which the World Health Assembly, concerned about the lack of attention to oral diseases, recognizes that oral health must be firmly integrated into the non-communicable diseases agenda and that oral health care interventions should be included in universal health coverage programs [1].

This is why, in this research, a detailed oral examination of the patients was carried out, and at the same time, the type of personality was analyzed by gender and age, in order to relate the personality traits and the oral health conditions that they presented, under the hypothesis that the oral health conditions would be related to the personality traits in adult patients who attended the CESS dentistry area. All of these results will allow us to guide more comprehensive, preventive, and/or curative interventions in oral health in the future.

## 2. Materials and Methods

A descriptive, observational, and correlational study was carried out in the area of dentistry at the Center for Health Studies and Services of the Universidad Veracruzana in the city of Veracruz, Veracruz, Mexico. The population of this study corresponded to patients who came for dental treatment. The sample size was estimated based on correlation studies considering a confidence level of 95% with a statistical power of 93%, based on a previous study [14] that found a correlation of 0.230 between personality traits and attitudes toward oral health, therefore leading to a resulting sample size of 180 people for this study. The following criteria were considered for patient selection. Inclusion: over 18 years of age, of any gender, who agreed to participate and signed the informed consent. Exclusion: patients who were under psychiatric treatment and pregnant women. Elimination: those who did not answer any question in the tests, who presented incomplete data, or if in the OHI-S review the patients did not have any of the six teeth used to carry out the index. It should be noted that 16 patients were eliminated for these reasons. The selected population signed an informed consent form after learning about the scientific objectives of the project, its technical basis, and the terms of confidentiality.

The protocol was reviewed and approved by the Research Committee of the Biological Research Institute of the Universidad Veracruzana (22-05) and the Ethical of Investigation Committee of the Psychological Research Institute of the Universidad Veracruzana (202408). All dental examinations were performed by two female dental surgeons (one of them specialized in rehabilitation and author of this article) trained to evaluate all indices. Examinations were performed in a dental unit with a light, mirror, and explorer, and with a stethoscope. To calibrate the examiners for the performance of the indices, random checks were performed on the patients by both examiners to identify intra-examiner errors.

### 2.1. Instruments for Data Collection

#### 2.1.1. Simplified Oral Hygiene Index (OHI-S)

With this index, the degree of oral hygiene can be determined. The mouth was divided into six parts (sextant) and six specific teeth were checked, one for each sextant. To review the teeth, it was required that they were completely erupted to adequately calculate the presence of dental plaque or calculus. Only six surfaces were evaluated, one from each selected tooth. The OHI-S has a minimum value of 0 and a maximum value of 6, accounting for dentobacterial plaque and calculus. The average obtained was identified with the parameters established according to the following scale: excellent oral hygiene 0, good 0.1–1.2, regular 1.3–3.0, and poor 3.1–6.0 [15].

#### 2.1.2. Caries Experience (DMFT Index)

This was developed by Klein, Palmer, and Knutson during a study of the dental status and treatment need of children attending elementary schools in Hagerstown, Maryland, USA, in 1935. It has become the fundamental index of dental studies carried out to quantify the prevalence of dental caries. It indicates the experience of both present and past caries, as it takes into account teeth with caries lesions and with previously performed treatments. It is obtained from the sum of the carious, lost, and filled permanent teeth, including the indicated extractions, among the total number of individuals examined, which is why it is an average. Only 28 teeth were considered, and for better analysis and interpretation, they had to be broken down into each of their parts and expressed in proportion or average. This is very important when comparing populations. The results could range from 0 to 28 and were expressed as follows: DMFT = 0–1.1 very low, 1.2–2.6 low, 2.7–4.4 moderate, 4.5–6.5 high, 6.6+ very high [16].

#### 2.1.3. Physical Examination of the Temporomandibular Joint (TMJ)

This was performed by palpation and auscultation of the TMJ to identify noises, pain, mandibular deviation, and limitation of movement. In this case, a review was conducted and it was determined whether the patient presented any of the aforementioned signs and symptoms (i.e., noises, pain, mandibular deviation, or limitation of movement). If the patient presented one to two signs, it was determined that he or she had a TMJ disorder. Thus, the results of this section are expressed as the presence/absence of TMJ disorder [17].

### 2.2. Identification of Parafunctional Habits

This was carried out through direct questioning of the patient, asking whether they had any of the following habits: biting their nails, sucking their thumb, constantly pushing their tongue toward their teeth, sleeping with their mouth open, or if they clenched or ground their teeth during the day or night. The results are given by whether the habit is present and which one, or if it is not present [18].

#### 2.2.1. Oral Health Impact Profile (OHIP)

This is one of the most widely used instruments for evaluating the perception of oral health in patients and its impact on daily quality of life. The questionnaire has seven domains: functional limitation, physical pain, psychological discomfort, physical disability, psychological disability, social disability, and handicap. Furthermore, it has been shown to be reliable, sensitive to change, and exhibit adequate cross-cultural internal consistency [19]. For the analysis of the overall levels of social impact, responses to individual questions from the seven OHIP-14 categories were standardized and summed to produce a single summary score. The coded responses for individual questions were rated on a Likert-type scale ranging from 0 for “never” to 4 for “almost always”. To obtain the score, the values are added, thus being able to have a minimum of 0 points and a maximum of 56 points for each patient. Therefore, low scores indicate better self-perceived quality of life and high scores indicate worse self-perceived quality of life [20]. Moreover, it can be expressed as negative and positive self-perception. This instrument was validated for the Spanish version for the Mexican population by Castejón-Pérez et al. [21], who obtained high values of internal consistency for the OHIP-Mx-49 (0.96), dimensions (0.79–0.86), repeatability in the instrument (0.877), and all dimensions, except for social disability (0.176).

#### 2.2.2. Courtauld Emotional Control Suppression Scale (CECS)

Prepared and validated by Watson and Greer and adapted and translated into Spanish by Durá et al. [22], this is the most relevant instrument in research on emotional suppression, as it evaluates the degree to which individuals claim to control their reactions when a negative emotion is experienced. The CECS is a questionnaire of 21 items separated into three subscales for the specific report of suppression, the expression of anger, anxiety, and depression. The response categories for each item are rated on a 4-point Likert-type scale ranging from “almost never” to “almost always”. Therefore, higher CECS scores correspond to higher levels of emotional suppression. The internal consistency of the CECS subscales was good: alpha coefficients ranged from 0.86 (anger subscale) to 0.88 (depressed mood and anxiety subscales); and the total CECS score was 0.95. Therefore, the reliability of the instrument was statistically satisfactory.

### 2.3. Big Five Inventory

Prepared by John et al. [23], this is a self-report measure that through 44 items, evaluates the five styles: extroversion, agreeableness, conscientiousness, neuroticism, and openness. This model assumes that personality consists of the individual, emotional, interpersonal, experiential, and motivational styles that make each person different from another. A Likert-type scale is used with five response options ranging from completely disagree to completely agree. This inventory was validated in the Mexican population by Chavira-Trujillo and Celis de la Rosa [24], and the results showed adequate reliability (α = 0.78) of the test. Furthermore, the evaluation of exploratory and confirmatory validity suggests that the instrument can be used in the Mexican population.

#### 2.3.1. Data Analyses

Descriptive statistics such as means, standard deviations, and proportions were performed to explore oral health conditions with respect to demographic variables. In turn, a correlation matrix was estimated between the entire set of variables under study. Likewise, a principal component analysis (PCA) was performed on the entire dataset to obtain a general overview of the data. All statistical analyses were carried out in R-project version 4.3.1 [25].

#### 2.3.2. Regression Analysis

The relationship between the total scores of the Simplified Oral Hygiene Index (OHI-S), average number of decayed, missing and filled teeth (DMFT), and oral health impact profile (OHIP) with respect to the demographic variables, emotional suppression, and personality traits were evaluated using multiple linear regression analysis. The model variables were transformed using natural logarithm for analysis. The relationship between the presence or absence of mandibular deviation (MD), joint noises (RA), non-carious lesions (NCL), and parafunctional habits (HPF) with respect to the demographic variables, emotional suppression, and personality traits was evaluated by the analysis with logistic regression.

#### 2.3.3. Network Analyses

Network analyses were conducted to examine and explain the relationships between the oral health index total scores, personality traits, and emotional suppression variables. Networks were estimated for the complete dataset by gender and by oral health condition. The variables under study were transformed using natural logarithm for analysis. For network estimation, a non-regularized model was selected using the ggmModelSelect function of the qgraph package of R [26], which searches for an optimal Gaussian graphical model (GGM) by adding and removing edges to the network until minimizing the extended Bayesian information criterion (EBIC).

In the graphical representation of the network, the color of the edges represents the direction of the relationship (i.e., red edges for negative correlation and green edges for positive correlation) [26]. The colors of the relationships between OHI-S, DMFT, and OHIP with other nodes in the network were inverted because lower values represent better health conditions. The nodes of the network represent the variables under study and the edges represent the relationships between the pairs of nodes adjusted by the influence of the other nodes in the network [27,28]. The intensity of the relationship between nodes is indicated by the thickness of the edge [29]. Analysis and visual representation of the networks were performed using the qgraph package of R-project [25].

#### 2.3.4. Centrality Analysis

Centrality indicates the importance of a node within the network. To evaluate the importance of the nodes in each of the networks, strength centrality was performed, which represents the connectivity of a node with the others in the network. Strength centrality, called degree centrality in unweighted networks [30], corresponds to the sum of all of the absolute values of the weights of the edges directly connected to a node [27]. Particularly, this centrality is usually more stable than other centrality indices in psychometric networks [27]. Centrality measures are presented as standardized values (z-score).

#### 2.3.5. Network Accuracy and Stability

The precision of the network was evaluated using resampling analysis to estimate the 95% confidence intervals (CI) for each edge of the network, where the width of the intervals indicates the precision of the edges [27]. Network stability was evaluated using the correlation stability coefficient (CS) [31] using non-parametric resampling with 1000 samples. This method evaluates the degree to which interpretations about the network structure are maintained as the sample size decreases. The CS represents the maximum proportion of cases that can be removed from the dataset to retain a correlation of at least 0.7 between the original centrality and that calculated in the resamples with a probability of 95%. The CS takes values between 0 (toral instability) and 1 (total stability); the literature suggests CS values greater than 0.25 (moderate stability) or preferably greater than 0.5 (strong stability). These analyses were performed using the R project bootnet package [27].

### 2.4. Comparisons Between Networks

The comparison of networks between gender and oral health conditions was carried out by testing the network invariance (M), global strength (S), and edge invariance using the R-project v4.3.2 “NetworkComparisonTest” (NCT) [32], employing 1000 iterations. The network invariant structure (M) evaluates whether the distribution of edge weights in the network is similar between groups. Global strength (S) assesses the degree to which the overall connectivity between nodes is similar across groups, comparing the absolute sum of all edge weights to measure the overall strength of the network. Finally, edge invariance evaluates the difference in the weights of particular edges between networks.

## 3. Results

### 3.1. Description of the Population

A total of 184 patients who attended the dentistry department in the period from October 2022 to May 2023 participated in the study, of which 59.78% were women and 40.21% men. The age of the population was 18 to 79 years, with the age range of 21–40 years being the most prevalent (48.37%).

### 3.2. Oral Health Conditions

When analyzing the percentages of non-carious lesions, 87.50% presented attrition, with women presenting this lesion in a higher percentage (89.09%) and people over 60 years of age (94.11%). Likewise, the examination had a normal mandibular opening represented in 95.65%. It should be noted that the remaining 4.34% who presented limitations on opening were entirely women. Regarding mandibular deviation, this occurred in 63.58%, with women again having the highest percentage (64.54%). Joint sounds only occurred in 50% of the total population. In addition, women (50.90%) and people between 41 and 60 years old (62.79%) presented higher percentages of joint sounds. The parafunctional habit with the highest percentage was onychophagia with 45.10%, with men (45.94%) and people aged 18 to 20 years (62.85%) having the greatest presence (Table 1).

With respect to oral hygiene, 70.10% had good oral hygiene, without significant differences between gender, however, significant differences were found by age range (*p* ≤ 0.01), showing lower average values of the oral hygiene index in the range from 18 to 20 years (0.52 ± 0.39) and higher values in people over 60 years (1.24 ± 1) (Table 2 and Supplementary Material S1).

Regarding caries history (DMFT) for this population, there was an average of 9.98, which according to the WHO severity scale, is in the very high range (very low 0.0–1.1, low 1.2–2.6, moderate 2.7–4.4, high 4.5–6.5, and very high +6.6). Furthermore, no significant differences were found between gender and age range; lower values were observed in the range of 18 to 20 years (7.80 ± 5.39) and higher values in those over 60 years of age (14.76 ± 5.61) (Table 2 and Supplementary Material S2).

### 3.3. Self-Perception of Oral Health

The oral health impact profile (OHIP) reflected low scores (14.34 ± 9.43), which implies having better self-perceived oral health. However, the highest scores were observed in women (15.20 ± 10.20), that is, a more negative self-perception of their oral health. According to age, it could be observed that the older the age, the worse the self-perception of oral health, with people over 60 years of age having the highest oral health perception scores (17 ± 6.31) (Table 2).

### 3.4. Personality Traits

The predominant personality trait in this population was openness with 75.54% (37.21 ± 5.15), followed by conscientiousness 11.95% (32.61 ± 4.44), and neuroticism with the lowest percentage of 1% (27.73 ± 4.16). It should be noted that there were only two women who presented this trait; no significant differences were found by gender and age range (Table 2 and Supplementary Material S1).

### 3.5. Emotional Suppression

The average global emotional suppression of the population (both men and women) was high. Furthermore, the subscales of anger suppression, depression, and anxiety presented mean medium values both overall and by gender and age range, without significant differences (Table 2).

#### Relationship of Oral Health Conditions with Personality Variables

**Figure 1:**
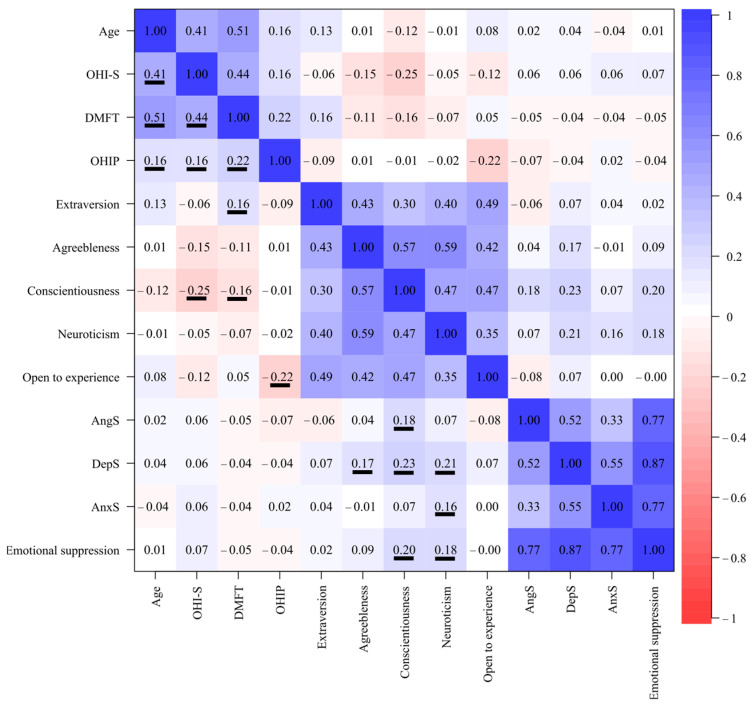
Pearson correlation between the oral health variables, personality traits, and emotional suppression. OHI-S: The Simplified Oral Hygiene Index; DMFT: average number of decayed, missing, and filled permanent teeth; OHIP: oral health impact profile; AngS: anger suppression, DepS: depression suppression; AnxS: anxiety suppression; S. Global: Global emotional suppression. Notes: Red shows the negative correlations and blue shows the positive ones. Variables with significant correlations are underlined.

As shown in Figure 1, the correlation analysis showed significant associations between the oral health condition variables and each other. Age had a high correlation coefficient with the DMFT (r = 0.51), medium with the OHI-S (r = 0.41), and low with the OHIP (r = 0.16). The DMFT and OHI-S had a correlation coefficient of 0.44, while the oral health self-perception (OHIP) correlated with the DMFT (r = 0.22) and with oral hygiene (OHI-S) (r = 0.16). Among the variables of emotional suppression and personality traits, significant associations could be seen between conscientiousness with the suppression of anger (r = 0.18) and depression (r = 0.23), while the personality trait neuroticism was correlated with the suppression of depression (r = 0.21). On the other hand, global suppression showed an association with the personality traits of conscientiousness (r = 0.20) and neuroticism (r = 0.18). Finally, significant relationships were found between the variables of oral health conditions and the personality traits, conscientiousness, and oral hygiene with a correlation coefficient of −0.25 and openness with the OHIP of −0.22.

**Figure 2:**
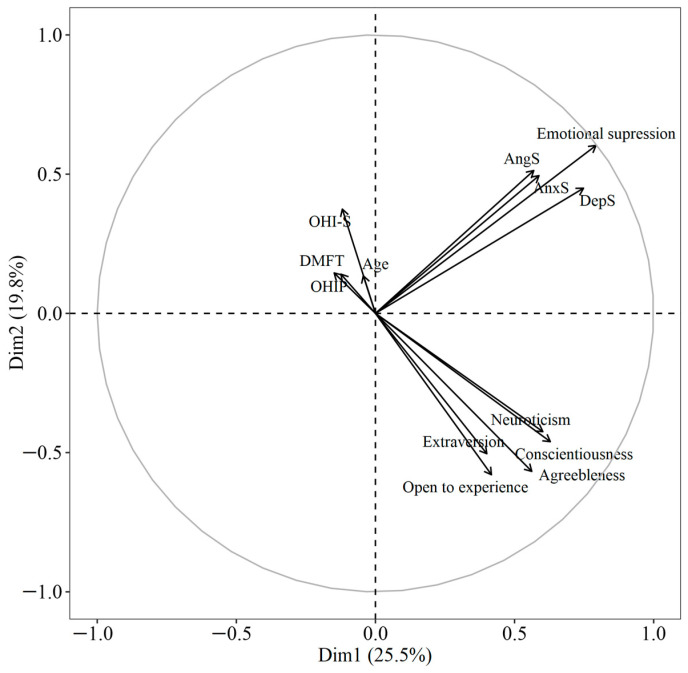
Principal component analysis. OHI-S: The Simplified Oral Hygiene Index; DMFT: average number of decayed, missing, and filled permanent teeth; OHIP: oral health impact profile; AngS: anger suppression; DepS: depression suppression; AnxS: anxiety suppression.

On the other hand, when performing a principal component analysis (PCA), the variables were grouped into two components that explained 44.52% of the variance: PC1 (27.10%), which included personality traits, and PC2 (19.8%), which included the variables of emotional suppression. It is worth noting that personality traits are inversely related to oral health conditions (Figure 2).

Additionally, several regression models were carried out to analyze the relationships with the various variables. In the multiple regression analysis of the oral health variables with respect to the demographic variables, personality trait, and emotional suppression, a significant relationship was observed for the caries index (DMFT), oral hygiene (OHI-S), and the perception of oral health (OHIP) with age, meaning that age influences the oral health conditions. On the other hand, the personality trait conscientiousness was related to oral hygiene index (OHI-S), predicting that oral hygiene is affected and/or benefited by this trait as well as the personality trait extroversion for the caries index (DMFT) and for oral health perception (OHIP) with the personality traits of openness and conscientiousness. In the case of emotional suppression, only anger suppression showed a negative effect for the OHIP (Table 3).

On the other hand, in the logistic regression analysis for non-carious lesions and mandibular deviation, age was the main direct association, and the personality traits agreeableness and openness were shown to be related to the presence of parafunctional habits. However, no association was found in joint sounds (Table 4).

**Figure 3:**
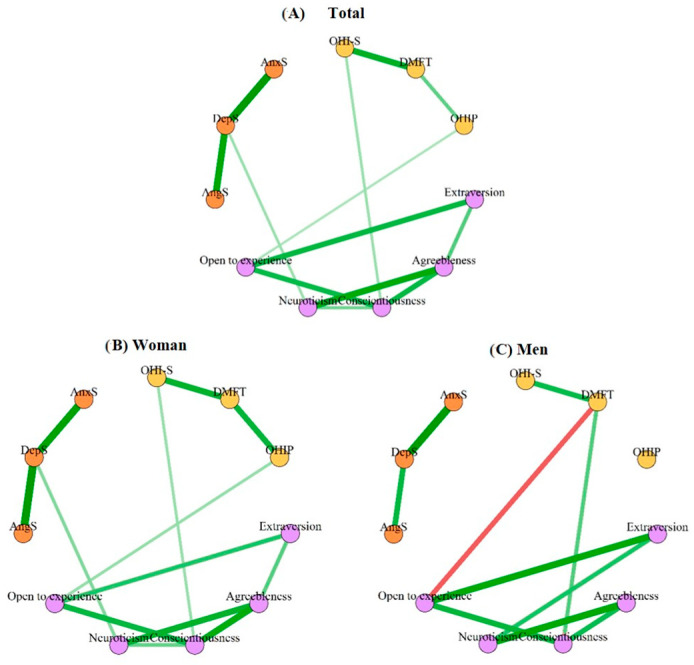
Network structure with variables of oral health conditions, personality traits and emotional suppression. Panel (**A**) complete network. (**B**) Women’s network. (**C**) Men’s network. OHI-S: The Simplified Oral Hygiene Index; DMFT: average number of decayed, missing, and filled permanent teeth; OHIP: oral health impact profile; AngS: anger suppression, DepS: depression suppression; AnxS: anxiety suppression. Note: The lilac nodes are personality traits, the orange nodes are subscales of emotional suppression, and the yellow nodes are indices of oral health. The green lines are positive correlations and the red lines are negative.

**Figure 4:**
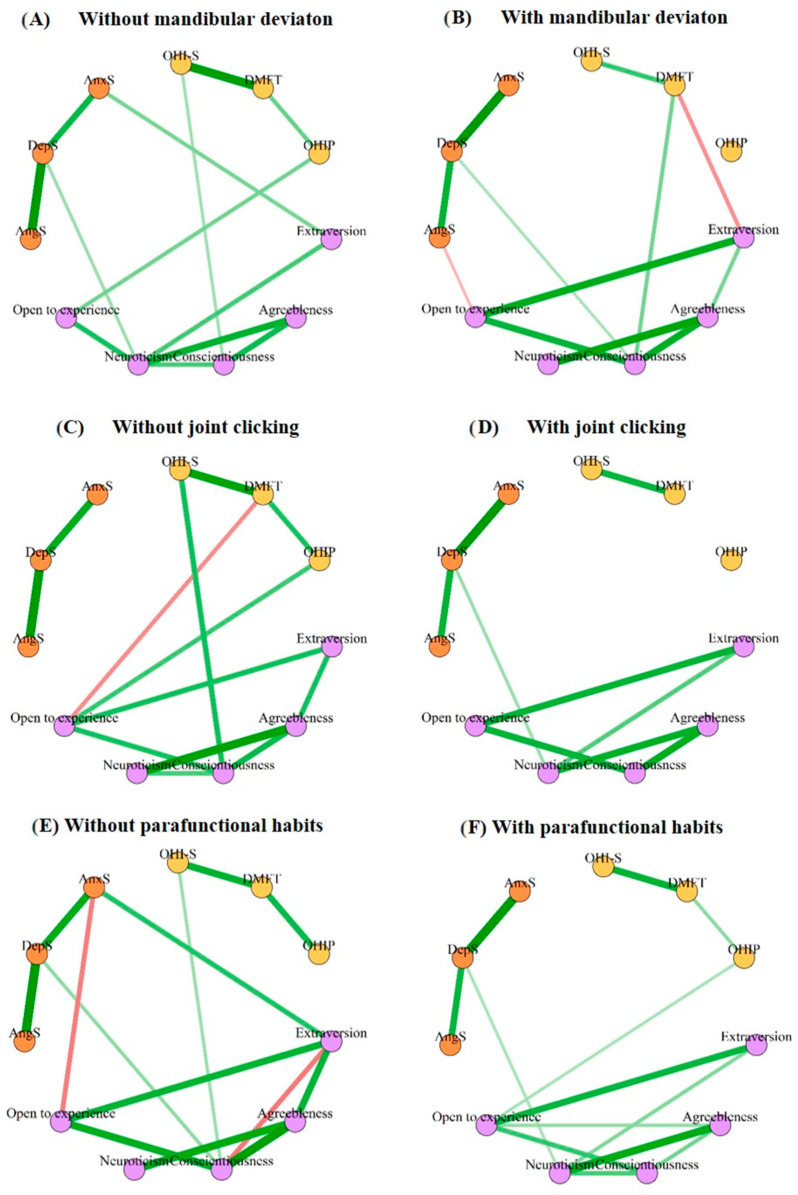
Network structure with variables of oral health conditions, personality traits and emotional suppression. (**A**) Network without mandibular deviation. (**B**) Network with mandibular deviation. (**C**) Network without joint noise. (**D**) Network with joint noise. (**E**) Network without parafunctional habits. (**F**) Network with parafunctional habits. OHI-S: The Simplified Oral Hygiene Index; DMFT: average number of decayed, missing, and filled permanent teeth; OHIP: oral health impact profile; AngS: anger suppression, DepS: depression suppression; AnxS: anxiety suppression. Note: The lilac nodes are personality traits, the orange nodes are subscales of emotional suppression, and the yellow nodes are indices of oral health. The green lines are positive correlations and the red lines are negative.

Finally, based on the correlations obtained between the studied variables (oral health conditions, personality traits, and emotional suppression), a network analysis was performed to compare the existing connections and their spatial distribution, considering gender and some oral pathologies (mandibular deviation, joint sounds, and parafunctional habits) (Figure 3 and Figure 4). The accuracy of the results was reliable due to the overlap of the sample results with the resampled results. In turn, the stability of the network was within reliable parameters (CS coefficient = 0.36), allowing valid results to be extracted (Supplementary Material S3).

Thus, when the connections and their distribution were analyzed both globally and by gender (Figure 3), we found that when disaggregating them into women and men (Panels B and C), these relationships varied significantly (Figure 1). For example, women (Figure 3B) presented a significant and positive relationship of the personality trait openness with self-perception of oral health, neuroticism with the suppression of depression, and conscientiousness with oral hygiene while men not only had fewer connections (i.e., the association of neuroticism with the suppression of depression and conscientiousness, of self-perception of oral health with the caries rate (DMFT), and of conscientiousness with oral hygiene was lost), but some connections changed, becoming negative (i.e., agreeableness with the caries index) or the variable with which it was connected changed (i.e., extroversion with neuroticism) (Figure 3C). That is, gender changes the way we react to life and therefore our health.

However, despite network comparisons by gender showing an invariant structure (M = 0.35, *p* = 0.57) and similar global strength (S = 0.16, *p* = 0.78), tests between specific edges revealed significant differences primarily between “DMFT”-“Conscientiousness” and “DMFT”-“Open to experience” (Supplementary Material S4).

On the other hand, when the networks were analyzed according to the absence or presence of certain oral pathologies (mandibular deviation, joint sounds, and parafunctional habits), the networks also presented relevant changes (Figure 4). For example, it is striking that many connections were lost when oral pathology was present. Particularly in the case of mandibular deviation, its presence generated negative connections of the openness trait with the suppression of anger and of the extroversion trait with the caries index (DMFT) (Figure 4A,B) while in the case of joint noises and parafunctional habits, their presence generated a profound disconnection from the network (Figure 4C–F). Multiple comparison tests showed invariant network structures for oral health conditions. Only the parafunctional habit networks were marginally significant in global strength (S = 1.21, *p* = 0.04) (Supplementary Material S4). However, tests between individual edges were significant between “OHI-S”-”DMFT”, “Neuroticism”-”Openness to experience”, and “Extraversion”-”AnxS” in the mandibular deviation networks, “Extraversion”-”AnxS” and “Openness to experience”-”AnxS” in the parafunctional habit networks, and “OHI-S”-”Conscientiousness” in the joint noise networks (Supplementary Material S5).

**Figure 5:**
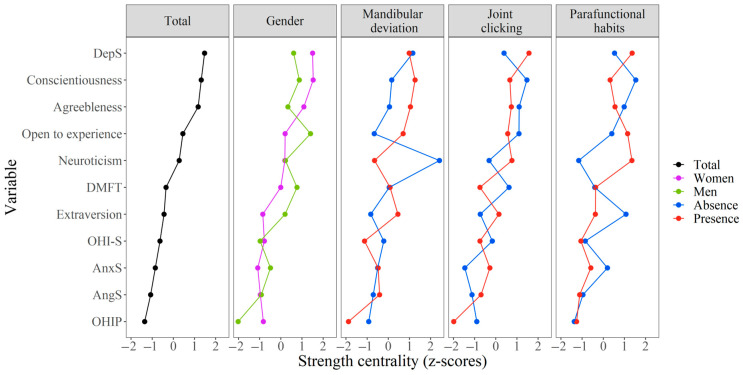
Strength centrality (standardized) of the oral health condition, personality traits, and emotional suppression variables of each estimated network. OHI-S: The Simplified Oral Hygiene Index; DMFT: average number of decayed, missing, and filled permanent teeth; OHIP: oral health impact profile; AngS: anger suppression, DepS: depression suppression; AnxS: anxiety suppression.

Likewise, it should be noted that when analyzing the centrality of the strength of each of the nodes in the network, not all nodes were equally important, neither in the total network, nor when disaggregated by gender or oral pathology (Figure 5). Thus, for example, it was observed that the suppression of depression, conscientiousness, and agreeableness maintained more important roles in the total network since they had strong relationships with other nodes within the network. This varied when analyzed by gender, since the nodes suppression of depression and awareness had a stronger weight in the women’s network while for men, it was the opening followed by the caries index (DMFT). Furthermore, when there was a presence of mandibular deviation, the consciousness node predominated; when there were joint noises, the suppression of depression node predominated; and when there was a presence of parafunctional habits, the suppression of depression and neuroticism predominated. It is important to note that when there was an absence of mandibular deviation, neuroticism was the most important node, and that when there were no parafunctional habits, it was the conscientiousness and extroversion nodes.

## 4. Discussion

Although oral diseases are mostly preventable, they are highly prevalent throughout the world, particularly in low- and middle-income countries. This is of great importance not only because they affect the population throughout their lives, causing discomfort, pain, deformities, and even death, but also because they impose a health, social, and economic burden in many countries [1].

This global lack of success in reducing oral disease is due to the lack of a comprehensive and collaborative approach between disciplines, subdisciplines, and professions in both clinical practice and research, a product of the biomedical/disease-centered reductionist model that remains dominant, resulting in physicians and dentists (including dental students and teachers) still lacking an individual-centered approach and a social dentistry perspective [33,34]. All of this has meant that fundamental aspects of health such as psychosocial aspects (i.e., personality, gender, age, among others) are left aside, since it is well-known that a person’s health, coping with illness, adherence to oral treatments, signs, and symptoms such as facets of wear and tear, temporomandibular joint dysfunction, and satisfaction and acceptance in prosthetic and orthodontic rehabilitation may be affected by their thoughts, emotions, and behaviors associated at their personality at age or gender [7,35,36,37,38,39,40]. This also means that basic science cannot be translated into clinical care, and in turn, a lack of public policies that achieve real advances in health, and therefore oral health and general health systems remain costly, fragmented, and ineffective [34].

Taking all of the above into account, in this research, in addition to carrying out a detailed oral examination of the patients, the personality type by gender and age was analyzed in order to guide more comprehensive, preventive, and/or interventions based on results in the future healing in oral health. This is especially in light of the fact that there have been few studies to review these aspects in dentistry, and that these vary in different populations and countries.

When analyzing the personality and emotional suppression in the patients who came for a check-up at the CESS dentistry clinic, we found, as expected, that oral pathologies, hygiene, caries index, and the self-perception of oral health changed depending on the patient trait and emotional profile. For example, through correlation and multiple regression analysis (Figure 1 and Table 3), we were able to observe that the personality traits extroversion, conscientiousness, and openness were significantly related to hygiene, caries rate, and self-perception of oral health, respectively. The observed negative relationship between hygiene and caries rate with conscientiousness was expected since this personality trait includes self-discipline, order, competition, striving for achievements, and reflection [41,42], thus these types of patients are expected to not only have good hygiene habits, but also know how to follow the dentist’s instructions and most likely go for check-ups very frequently, decreasing the probability of cavities [42]. On the contrary, extroversion had a positive relationship with the caries rate, similar to what was found by Gupta and Shetty [43], which could be due to the fact that people with this personality type visit the dentist less [6]. Finally, the trait of openness, related to intellect or discernment, actions, ideas, values, and having greater adaptability and mental flexibility [41,44], correlated with self-perception of health, which is similar to what was previously reported by Zakershahrak and Brennan [44], who not only related it to emotional stability, but this same effect seemed to reduce the adverse effects of health inequities. It should be noted that in the network analyses with and without oral pathologies, when there was a presence of mandibular deviation, joint sounds, and functional habits, the positive relationship between awareness and oral hygiene was lost (Figure 3). Therefore, the trait of conscientiousness was not only less present in temporomandibular disorders [45], but its absence had repercussions on oral hygiene. Likewise, certain personality traits tend to suppress certain emotions more, which in turn can indirectly affect oral health. Such is the case of agreeableness and neuroticism, which correlated with the suppression of depression, conscientiousness with the suppression of anger, and conscientiousness and neuroticism with global emotional suppression (Figure 1), and the network analyses in the patients with mandibular deviation and parafunctional habits presented a positive relationship with the suppression of depression and neuroticism. Furthermore, when there were parafunctional habits, the negative or regulatory relationship of the personality trait openness with the suppression of anger was lost (Figure 3). It should be noted that, although emotional suppression did not correlate with any oral health variable, in the multiple regression model, anger suppression was associated with a worse self-perception of oral health. Furthermore, although suppression was not directly related, it may have an indirect effect on oral health through personality traits. That is, personality serves as a bridge or connector between emotional suppression and oral health. Therefore, as reported by various authors, emotional suppression and repression have repercussions on health [46,47,48,49].

On the other hand, not only do personality and poor emotional management have repercussions on oral health. Our analyses showed that there were significant associations with age and gender. For example, age showed associations in both the correlation analysis and the multiple regression with hygiene, caries index, and self-perceived health (Figure 1 and Table 4), was grouped with these variables in the principal components (Figure 2), and showed a positive effect in the logistic regression analysis with non-carious lesions and mandibular deviation. All of these data, although not surprising due to the natural history of oral diseases and because it is one of the social determinants [50,51], do allude to the fact that health promotion and disease prevention programs are failing in our country. In addition, they point to some aspects that can be worked on in health education interventions. For example, in dental offices and health centers, it would be convenient for dentists, in addition to carrying out a dental clinical history, to apply psychometric instruments to diagnose personality types, emotional disorders, and stress and identify risk factors by age and gender. With these data, the dentist could guide the patient to improve their oral and behavioral habits as well as refer them to a multidisciplinary team for their education, and if necessary, for their care. This care model has been previously proposed by Barranca-Enriquez and Romo-González (2022) and includes not only patient education, but also the comprehensive and multidisciplinary training of dental professionals. Last, but not least, our work makes it clear that there is a need for analyses with a gender perspective, since women and men do not present the same susceptibilities or health risk behaviors. Particularly, women presented more attrition, more limitations in opening, deviations, and jaw noise than men. Furthermore, in the network analysis, women presented associations of neuroticism with the suppression of depression, which could perhaps explain the greater presence of mandibular anomalies. Although in the case of women, they presented relationships between the personality traits of conscientiousness and hygiene and self-perception of oral health with the trait of openness. In this regard, Lin et al. [52] found that women perceived poorer oral health more frequently compared to men, especially in terms of physical pain and psychological disability. According to these same authors, these results are expected since women aspire to live more comfortably and place more emphasis on their esthetic appearance than men, but their confidence could be affected only by a slight misalignment. On the other hand, in the case of the network in men, the caries rate was positively associated with the conscientiousness trait and negatively with openness. That is, dental practice and programs for the prevention of oral disease require a comprehensive view with a gender perspective, already previously proposed in various care models [3,33,53].

All of these results are of great relevance; however, it is important to highlight some limitations in the development of this research. Although the sample size was calculated from the correlations found for the personality variables and oral health conditions [14], given that the sample included patients who came for a consultation at the CESS dentistry unit, this generated a bias in the proportions of patients according to personality type. Thus, for example, out of 184 participants, only two had a high score in neuroticism, and these were also women. Therefore, previously reported associations of neuroticism with oral diseases and health risk behaviors have not been widely explored. Therefore, it is necessary to carry out studies in which the number of participants is increased, and that they are from the community in general and not patients who attend dental consultations.

## 5. Conclusions

The high prevalence of oral diseases in the world continues to be a public health problem due to the fact that research, the health system, and private clinical practice continue to focus on the remediation and rehabilitation of the disease, and in dental specialties (endodontics, periodontics, oral rehabilitation, etc.), this does not include comprehensive care with other health disciplines such as physicians, nutritionists, psychologists, chemists, nurses, etc.

In this regard, in this article, we show that oral pathologies, hygiene, caries index, and the self-perception of oral health change depending on the patient’s personality trait, emotional profile, gender, and age, aspects that are useful to identify the risk and protection factors, which can be communicated and educated in patients for their self-knowledge and self-care.

Likewise, the identification of these factors in the population can guide protocols for care and health promotion that are included in new public policies at the national and international levels.

All of this is important not only in the field of oral health, since the relationship between oral health and general health is well-known, but so that the reduction in oral diseases will simultaneously improve the epidemiological panorama, quality of life, and its economic impact on the population and health systems.

## Figures and Tables

**Table 1:** Frequencies and percentages of oral pathologies by gender and age.

			Gender				Age Range							
Oral Diseases	Total (n = 184)		Women (n = 110)		Men (n = 74)		18–20 (n = 35)		21–40 (n = 89)		41–60 (n = 43)		Over 60 (n = 17)	
Non-Carious Lesions	f	%	f	%	f	%	f	%	f	%	f	%	f	%
Attrition	**161**	**87.50%**	**98**	**89.09%**	63	85.13%	23	65.71%	82	92.13%	40	93.02%	**16**	**94.11%**
No injury	21	11.41%	12	10.90%	9	12.16%	12	34.28%	7	7.86%	2	4.65%	0	0.00%
Abrasion	7	3.80%	3	2.72%	4	5.40%	0	0.00%	3	3.37%	1	2.32%	3	17.64%
Abfraction	4	2.17%	0	0.00%	4	5.40%	0	0.00%	0	0.00%	4	9.30%	0	0.00%
Erosion	2	1.08%	1	0.90%	1	1.35%	0	0.00%	1	1.12	0	0.00%	1	5.88%
**Maximum opening**														
Normal +40 mm	**176**	**95.65%**	102	92.72%	74	100%	33	94.28%	84	94.38%	42	97.67%	17	100%
Limitation −40 mm	8	4.34%	**8**	**7.27%**	0	0.00%	**2**	**5.71%**	5	5.61%	1	2.32%	0	0.00%
**Mandibular deviation**														
With deviation	**117**	**63.58%**	**71**	**64.54%**	46	62.16%	19	54.28%	53	59.55%	32	74.41%	**13**	**76.47%**
No deviation	67	36.41%	39	35.45%	28	37.83%	16	45.71%	36	40.44%	11	25.58%	4	23.52
**Joint clicking**														
With noise	92	50.00%	**56**	**50.90%**	36	48.64%	12	34.28%	44	49.43%	**27**	**62.79%**	9	52.94%
Without noise	92	50.00%	54	49.09%	38	51.35%	**23**	**65.71%**	45	50.56%	16	37.20%	8	47.05%
**Parafunctional habits**														
No habit	**63**	**34.24%**	37	33.63%	**26**	**35.13%**	11	31.42%	26	29.21%	18	41.86%	**8**	**47.05%**
Onychophagia	**83**	**45.10%**	49	44.54%	**34**	**45.94%**	**22**	**62.85%**	44	49.43%	12	27.90%	5	29.41%
Mouth breathing	44	23.91%	27	24.54%	17	22.97%	9	25.71%	20	22.47%	11	25.58%	4	23.52%
Bruxism	32	17.39%	17	15.45%	15	20.27%	6	17.14%	17	19.10%	8	18.60%	1	5.88%
Digital suction	4	2.17%	3	2.72%	1	1.35%	0	0.00%	4	4.49%	0	0.00%	0	0.00%
Atypical swallowing	1	0.54%	0	0.00%	1	1.35%	0	0.00%	1	1.12%	0	0.00%	0	0.00%

Note: High values in each condition are highlighted in bold.

**Table 2:** Means and standard deviation of the oral conditions, personality, and emotional suppression by gender and age.

			Gender				*p*	Age Range						*p*
Variables	Total		Women		Men			18–20		21–40		41–60		
	Mean	SD	Mean	SD	Mean	SD		Mean	SD	Mean	SD	Mean	SD	
OHI-S	0.77	0.79	0.74	0.83	0.82	0.75	0.28 ^a^	0.52	0.39	0.59	0.65	1.16	0.99	<0.01 ^b^
DMFT	9.98	5.40	10.00	5.60	9.95	5.13	0.98 ^a^	7.80	5.39	8.20	4.55	13.53	3.91	<0.01 ^b^
OHIP	14.34	9.43	15.20	10.20	13.05	8.05	0.18 ^a^	13.74	9.67	13.10	9.79	16.33	9.22	0.10 ^b^
Extraversion	28.67	4.31	28.84	4.47	28.43	4.09	0.48 ^a^	27.49	5.16	28.54	3.45	29.74	5.00	0.09 ^b^
Agreeableness	29.95	4.46	30.21	4.53	29.57	4.35	0.22 ^a^	29.06	3.74	30.20	4.26	30.26	5.09	0.47 ^b^
Conscientiousness	32.61	4.44	32.54	4.39	32.73	4.55	0.84 ^a^	32.26	4.67	33.17	3.64	32.42	4.78	0.37 ^b^
Neuroticism	27.73	4.16	28.06	4.33	27.24	3.88	0.21 ^a^	26.60	4.35	28.01	3.86	28.49	4.31	0.13 ^b^
Open to experience	37.21	5.15	36.58	5.07	38.15	5.14	0.06 ^a^	35.86	5.17	37.29	4.76	38.30	5.10	0.16 ^b^
Anger S.	17.79	3.78	18.07	3.96	17.38	3.49	0.25 ^a^	17.74	3.88	17.87	3.59	17.53	3.88	0.84 ^b^
Depression S.	18.41	3.96	18.09	3.81	18.89	4.15	0.3 ^a^	18.20	4.03	18.31	3.69	18.81	4.41	0.90 ^b^
Anxiety S.	18.31	3.57	17.82	3.59	19.04	3.44	0.06 ^a^	18.63	3.81	18.35	3.71	18.16	3.54	0.92 ^b^
Emotional Suppression	54.51	9.09	53.98	9.25	55.31	8.88	0.47 ^a^	54.57	9.51	54.53	8.80	54.51	9.41	0.98 ^b^

Note: DMFT: mean number of decayed, missing, and filled permanent teeth; OHI-S: The Simplified Oral Hygiene Index; OHIP: oral health impact profile; Ang S.: anger suppression, Dep S.: depression suppression; Anx S.: anxiety suppression. ^a^ Wilcoxon rank sum test, ^b^ Kruskal–Wallis. High and/or significant values in each condition are highlighted in bold.

**Table 3:** Multiple regression analysis of the oral health variables with respect to demographic and psychological variables.

	DMFT (R^2^ = 0.30)			OHI-S (R^2^ = 0.22)			OHIP (R^2^ = 0.14)		
Variable	Β	EE	T	Β	EE	t	β	EE	t
Intercept	0.48	11.84	0.04	0.42	0.38	1.09	2.53	1.07	2.37
Gender	−0.51	0.74	−0.69	0.02	0.02	0.81	−0.06	0.07	−0.84
Age	13.84	1.87	7.40 ***	0.35	0.06	5.78 ***	0.62	0.17	3.70 ***
Extroversion	12.16	5.97	2.04 *	−0.11	0.19	−0.57	−0.11	0.54	−0.20
Agreeableness	−10.90	7.19	−1.51	−0.13	0.23	−0.55	−0.12	0.65	−0.19
Conscientiousness	−5.40	7.68	−0.70	−0.49	0.25	−1.99 *	1.80	0.69	2.58 *
Neuroticism	−4.15	6.79	−0.61	0.23	0.22	1.05	−0.78	0.61	−1.28
Open to experience	3.49	7.09	0.49	−0.09	0.23	−0.41	−1.92	0.64	−2.99 **
AngS	−1.88	4.36	−0.43	0.06	0.14	0.44	−0.88	0.39	−2.22 *
DepS	−0.03	4.71	−0.01	0.07	0.15	0.49	−0.23	0.43	−0.53
AnxS	−0.95	4.85	−0.19	−0.01	0.16	−0.05	0.62	0.44	1.42

Note: * *p* < 0.05; ** *p* < 0.01; *** *p* < 0.001; DMFT: mean number of decayed, missing, and filled permanent teeth; OHI-S: The Simplified Oral Hygiene Index; OHIP: oral health impact profile; AngS: anger suppression, DepS: depression suppression; AnxS: anxiety suppression. High and/or significant values in each condition are highlighted in bold.

**Table 4:** Logistic regression analysis of the variables of non-carious lesions, mandibular deviation, joint clicking, and parafunctional habits with respect to the demographic variables, personality traits, and emotional suppression.

	NCCL			MD			JC			PH		
Variable	β	EE	z	β	EE	z	β	EE	z	Β	EE	z
Intercept	4.90	3.19	1.53	0.78	1.82	0.43	2.01	1.74	1.15	2.30	1.89	1.22
Gender	−0.10	0.54	−0.19	−0.19	0.35	−0.55	−0.17	0.33	−0.50	0.31	0.37	0.85
Age	0.07	0.03	2.71 *	0.02	0.01	2.05 *	0.01	0.01	1.30	−0.02	0.01	−1.65
Extroversion	0.10	0.08	1.38	0.03	0.05	0.57	0.01	0.04	0.26	−0.06	0.05	−1.22
Agreeableness	−0.11	0.09	−1.21	0.09	0.05	1.66	−0.02	0.05	−0.40	0.11	0.05	2.06 *
Conscientiousness	−0.10	0.08	−1.23	−0.05	0.05	−0.91	0.03	0.05	0.67	0.06	0.05	1.07
Neuroticism	0.14	0.08	1.66	−0.04	0.05	−0.75	−0.04	0.05	−0.93	0.02	0.05	0.37
Open to experience	−0.08	0.06	−1.27	−0.06	0.04	−1.46	−0.06	0.04	−1.58	−0.10	0.04	−2.40 *
AngS	0.01	0.08	0.14	−0.08	0.05	−1.54	−0.04	0.05	−0.83	0.00	0.05	0.03
DepS	−0.03	0.09	−0.32	0.05	0.06	0.97	0.05	0.05	0.97	0.00	0.06	−0.03
AnxS	−0.10	0.09	−1.11	0.06	0.06	1.06	0.02	0.05	0.32	−0.08	0.06	−1.43

Note: * *p* < 0.05. NCCL: non-carious lesions; MD: mandibular deviation; JC: joint clicking; PH: parafunctional habits; AngS: anger suppression, DepS: depression suppression; AnxS: anxiety suppression. High and/or significant values in each condition are highlighted in bold.

## Data Availability

All of the data are available at https://github.com/ihuesca/Database_OralHealth.

## Supplementary Materials

The following supporting information can be downloaded at: https://www.mdpi.com/article/10.3390/dj12120391/s1, Supplementary Material S1. Frequencies and percentages of the oral indexes and the personality profile. Supplementary Material S2. Frequencies and percentages of the DMFT index disaggregated by Decayed, Missing, and Filled Permanent Teeth. Supplementary Material S3. Stability and precision of the estimated network. (A) Stability of strength centrality of network. (B) Precision of network edges. Supplementary Material S4. Network comparison test (NCT) between gender and oral health conditions. Supplementary Material S5. Network comparison test (NCT) on individual edges.
