# Supplementary material for: Variations in Some Features of Oral Health by Personality Traits, Gender, and Age: Key Factors for Health Promotion
Source: Dent J (Basel). 2024 Dec 3;12(12):391. doi: 10.3390/dj12120391 (PMC11674120; doi:10.3390/dj12120391)
Supplement: Supplementary file 1 [file dentistry-12-00391-s001.zip › dentistry-3294260-supplementary.pdf]

Supplementary materials

Supplementary material 1. Frequencies and percentages of the oral indexes and the personality profile

			Gender				Age ranges							
**Indexes**	**Total (n=184)**		**Women (n=110)**		**Men (n=74)**		**18-20 (n=35)**		**21-40 (n=89)**		**41-60 (n=43)**		**Over 60 (n=17)**	
**OHI-S**	**f**	**%**	**f**	**%**	**f**	**%**	**f**	**%**	**f**	**%**	**f**	**%**	**f**	**%**
Excellent	27	14.67%	18	16.36%	9	12.16%	7	20.00%	19	21.34%	1	2.32%	0	0.00%
Good	129	70.10%	80	72.72%	49	66.21%	28	80.00%	59	66.29%	29	67.44%	13	76.47%
Regular	24	13.04%	9	8.18%	15	20.27%	0	0.00%	10	11.23%	11	25.58%	3	17.64%
Poor	4	2.17%	3	2.72%	1	1.35%	0	0.00%	1	1.12%	2	4.65%	1	5.88%
**DMFT**														
Without cavities	10	5.43%	6	5.45%	4	5.40%	4	11.42%	4	4.49%	2	4.65%	0	0.00%
Very low	4	2.17%	3	2.72%	1	1.35%	1	2.85%	3	3.37%	0	0.00%	0	0.00%
Low	19	10.32%	14	12.72%	5	6.76%	3	8.57%	12	13.48%	2	4.65%	2	11.76%
Moderate	23	12.50%	11	10.00%	12	16.21%	6	17.14%	14	15.73%	2	4.65%	1	5.88%
High	127	69%	76	69.09%	51	68.91%	21	60%	55	61.79%	37	86.04%	14	82.35%
Very high	1	1%	0	0%	1	1.35%	0	0%	1	1.12%.	0	0.00%	0	0.00%
**OHIP**														
0	20	10.86%	12	10.90%	8	10.81%	5	14.28%	14	15.73%	1	2.32%	0	0.00%
1-10	46	25.00%	23	21%	23	31.08%	9	25.71%	22	24.71%	12	27.90%	3	17.64
11-20	76	41.30%	47	42.72%	29	39.18%	14	40.00%	34	38.20%	18	41.86%	10	58.82%
21-30	33	17.93%	20	18.18%	13	17.56%	5	14.28%	15	16.85%	9	20.93%	4	23.52%
31-40	9	4.89%	8	7.27%	1	1.35%	2	5.71%	4	4.49%	3	6.97%	0	0.00%
41-56	0	0.00%	0	0.00%	0	0.00%	0	0	0	0.00%	0	0.00%	0	0.00%
**Personality profile**														
Open to experience	139	75.54%	78	70.90%	61	82.43%	23	65.71%	69	77.52%	32	74.41%	15	88.23%
Concientiounsness	22	11.95%	14	12.72%	8	10.81%	5	14.28%	12	13.48%	4	9.30%	1	5.88%
Concientiounsness - Open to experience	9	4.89%	7	6.36%	2	2.70%	2	5.71%	4	4.49%	3	6.97%	0	0.00%
Extraversion	4	2.17%	3	2.72%	1	1.35%	1	2.85%	0	0.00%	2	4.65%	1	5.88%
Agreebleness	4	2.17%	4	3.63%	0	0.00%	1	2.85%	3	3.37%	0	0.00%	0	0.00%
Agreebleness - Open to experience	4	2.17%	2	1.81%	2	2.70%	2	5.71%	1	1.12%	1	2.32%	0	0.00%
Neuroticism	2	1.08%	2	1.81%	0	0.00%	1	2.85%	0	0.00%	1	2.32%	0	0.00%

Supplementary material 2. Frequencies and percentages of the DMFT index disaggregated by Decayed, Missing, and Filled Permanent Teeth

**Total (n=184)**					**Women (n=110)**					**Men (n=74)**				
D	M	F	TOTAL	DMF	D	M	F	TOTAL	DMF	D	M	F	TOTAL	DMF
870	235	731	1836	9.98	541	134	425	1100	10	329	101	306	736	9.95

**18-20 (n=35)**					**21-40 (n=89)**					**41-60 (n=43)**					**mas 60 (n=17)**				
D	M	F	TOTAL	DMF	D	M	F	TOTAL	DMF	D	M	F	TOTAL	DMF	D	M	F	TOTAL	DMF
182	5	77	273	7.8	383	28	319	730	8.2	251	103	228	582	13.53	54	99	98	251	14.76

Supplementary Material 3. Stability and precision of the estimated network. A) Stability of strength centrality of network, B) Precision of network edges.

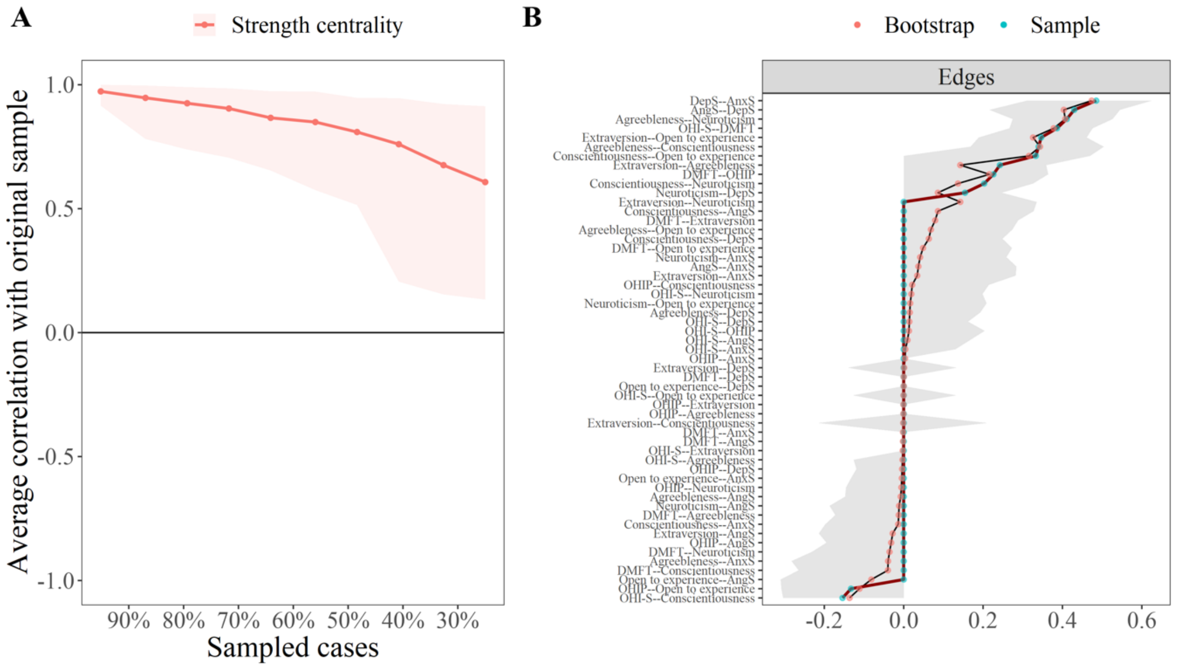

Supplementary Material 4. Network comparison test (NCT) between gender and oral health conditions.

**Group**	**[a]M**	**P-value**	**[b]S**	**P-value**
Gender	0.356	0.571	0.167	0.788
Mandibular desviation	0.413	0.419	0.390	0.547
Joint clicking	0.287	0.901	0.667	0.255
Oral parafunctions	0.379	0.57	1.210	0.049

[a]Network invariance test; [b]Test of global centrality weights.

Supplementary Material 5. Network Comparison Test (NCT) on individual edges.

**Group**	**Nodes between edges**		**Statistic[a]**	**P-value**
Gender	DMFT	Conscientiousness	0.263	0.027
Gender	DMFT	Open to experience	0.341	0.008
Mandibular desviation	OHI-S	DMFT	0.246	0.028
Mandibular desviation	Neuroticism	Open to experience	0.323	0.007
Mandibular desviation	Extroversion	AxnS	0.225	0.033
Oral parafunctions	Extroversion	AxnS	0.293	0.020
Oral parafunctions	Open to experience	AxnS	0.264	0.004
Joint clicking	OHI-S	Conscientiousness	0.288	0.032

Note: Only significant edges at $p<0.05$ are shown. [a]Test statistic from Network Comparison Test (NCT).
